# Metformin Use and Long-term Outcomes Including Aneurysm Sac Dynamics Following EVAR for Infrarenal Abdominal Aortic Aneurysm: “A Retrospective Study”

**DOI:** 10.1177/15266028241268500

**Published:** 2024-08-14

**Authors:** Olivier L. R. M. van Tongeren, Vinamr Rastogi, David E. Vecht, Klaas H. J. Ultee, Sanne E. Hoeks, Hence J. M. Verhagen, Jorg L. de Bruin

**Affiliations:** 1 Department of Vascular Surgery, Erasmus Medical Centre, Rotterdam, The Netherlands; 2 Department of Vascular and Endovascular Surgery, Beth Israel Deaconess Medical Center, Boston, MA, USA; 3 Department of Anesthesiology, Erasmus Medical Centre, Rotterdam, The Netherlands

**Keywords:** EVAR, metformin, aneurysm sac, mortality, outcomes

## Abstract

**Purpose:**

Metformin, widely used for the treatment of diabetes mellitus (DM), has shown potential for inhibiting abdominal aortic aneurysm (AAA) growth by reducing extracellular matrix remodeling and inflammation. However, its influence on clinical outcomes and aneurysm sac dynamics after endovascular aneurysm repair (EVAR) remains uncertain. This retrospective study aims to explore the effects of metformin on long-term outcomes following EVAR.

**Materials and Methods:**

Patients who underwent elective standard EVAR for infrarenal AAA at a single academic Dutch hospital from 2000 to 2022 were included. We collected baseline patient demographics, comorbid conditions, anatomical and operative characteristics, and 30-day postoperative events. Metformin use was defined as using it preceding EVAR. The primary outcome, the postoperative aneurysm sac volume over time, was investigated using linear mixed-effects modeling. The secondary outcomes, 8-year all-cause mortality and freedom from graft-related events, were evaluated using Kaplan-Meier methods.

**Results:**

We analyzed 685 patients, including 634 (93%) non-metformin users and 51 (7%) metformin users. The median follow-up period was similar (4.0 years [IQR=1.5, 6.5] vs 5.0 years [IQR=2.0, 8.0]; p=0.091). Patients on metformin had a preoperative aneurysm sac volume of 153 cc (IQR=114, 195) compared with 178 cc (IQR=133, 240) for non-metformin patients (p=0.054). At 30 days post-EVAR, metformin patients had a comparable mean aneurysm sac volume compared with non-metformin patients (metformin: –19.4 cc [95% confidence interval [CI]: –47.4, 8.5]; p=0.173). The effect of metformin on aneurysm growth over time was not significant (–3.9 cc/year; [95% CI: –22.7, 14.9]; p=0.685). Following risk-adjusted analysis, metformin use was associated with similar rates of all-cause mortality (metformin vs no metformin: 50% vs 44%; hazard ratio [HR]=1.11, 95% CI: 0.66, 1.88; p=0.688) and freedom from graft-related events (metformin vs no metformin: 63% vs 66%; HR=1.82, 95% CI: 0.98, 3.38; p=0.059).

**Conclusion:**

Although metformin use may reduce preoperative AAA growth, it does not seem to influence overall/long-term post-EVAR AAA sac dynamics, all-cause mortality, or freedom from graft-related events. These findings suggest that the potential protective effect of metformin on AAA may not be sustained after EVAR. Further prospective studies are needed to investigate the mechanisms underlying the potential role of metformin in AAA management following EVAR.

**Clinical Impact:**

There is currently no approved pharmacological treatment available to slow the abdominal aortic aneurysm (AAA) growth rate and reduce the related risk of rupture. In our retrospective analysis including 685 patients undergoing EVAR for infrarenal AAA, we found that metformin use was not associated with improved post-EVAR outcomes, such as a reduction of aneurysm sac volume over time, eight-year all-cause mortality, or freedom of graft-related events. These findings suggest that the potential protective effect of metformin on AAA may not be sustained after EVAR and underscore the need for ongoing research into this area.

## Introduction

There is currently no approved drug therapy available to mitigate abdominal aortic aneurysm (AAA) growth and the associated risk of rupture.^1,2^ The pathogenesis of AAA development is multi-factorial, encompassing loss of vascular matrix and infiltration of inflammatory cells.^3^ Conversely, excessive accumulation of vascular matrix is a hallmark of diabetes mellitus (DM).^3^ Although DM is widely recognized as a major cardiovascular risk factor, a paradoxical inverse association between the presence of DM and the prevalence and growth of AAA has been observed.^3^ The exact underlying mechanism of this association remains elusive.^3^ Some researchers have proposed that the increased vascular matrix seen in DM may compensate for the matrix loss associated with AAA,^3,4^ while others have suggested that the pleiotropic effects of medications prescribed to diabetic patients may be involved.^1,3,5^ The evidence that DM is associated with decreased growth rate of AAA, may result in a lower rate of sac growth following endovascular aneurysm repair (EVAR), in a lower need for reinterventions following EVAR.^6^

A literature review has suggested that antidiabetic medications may provide direct inhibitory effects on the pathology of AAA.^3^ Among these medications, metformin is the most widely prescribed drug for DM globally.^5^ Evidence suggests that metformin may impact the progression of AAA through multiple mechanisms, including reduction of extracellular matrix remodeling and exerting anti-inflammatory effects.^1^ The use of metformin may result in a lower prevalence of AAA and limit its growth rate.^5,7^ Numerous ongoing clinical trials are currently investigating the use of metformin in non-DM patients prior to surgery.^8,9^ Nevertheless, the existing literature lacks sufficient evidence regarding the influence of metformin on clinical outcomes and aneurysm sac dynamics after EVAR.

Given the evidence of its potential to inhibit the pathology of AAA, metformin appears to be a promising candidate drug for limiting its growth. This study aims to explore the association between preoperative use of metformin and aneurysm sac dynamics and long-term outcomes following infrarenal EVAR.

## Materials and Methods

### Study Design and Patient Population

**Figure 1.**
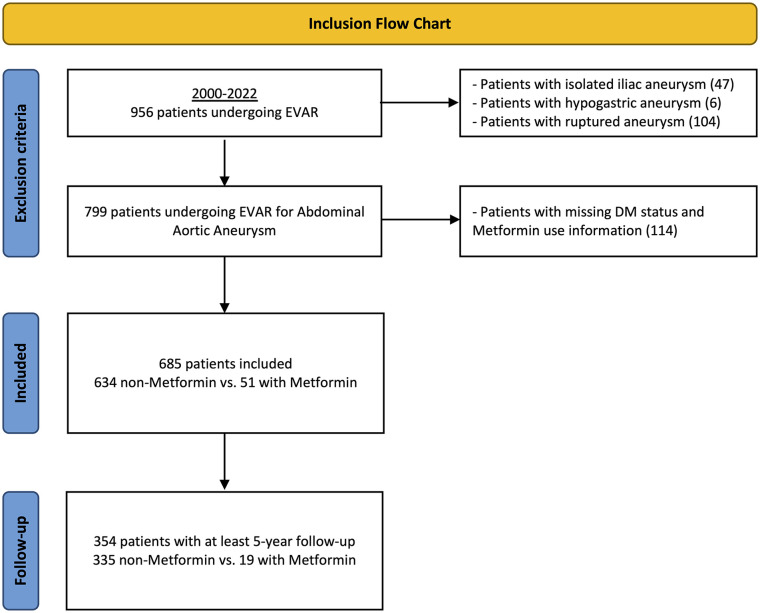
Inclusion diagram. DM, diabetes mellitus; EVAR, endovascular aneurysm repair.

A retrospective study was conducted on all consecutive patients who underwent elective standard infrarenal EVAR for AAA at a single academic hospital in the Netherlands between 2000 and 2022 (n=956; Figure 1). Our study population inherently did not include patients who underwent complex EVAR procedures or those who received adjunctive interventions, such as the application of endoanchors or the use of iliac branched devices. Patients with isolated iliac (n=47), hypogastric (n=6), or ruptured aneurysms (n=104) were excluded from this study. Furthermore, patients with missing DM status and missing perioperative metformin use information (n=114) were also excluded from analysis. The study protocol was approved by the hospital’s institutional and ethical review board, which waived the requirement for informed consent due to the retrospective design of the study (MEC-2022-0591). This study adhered to the STROBE (Strengthening The Reporting Observational Studies in Epidemiology) criteria to ensure proper reporting of observational studies.^10^

### Data Collection and Image Measurements

Baseline patient demographics, comorbid conditions, anatomical and operative characteristics, postoperative events within the first 30 days, as well as long-term outcomes, including survival and all relevant details were gathered for each patient from the hospital’s electronic patient database (ChipSoft-HIX, Amsterdam, The Netherlands). This data collection process was performed by an independent observer, who was blinded to the study outcomes, in accordance with the recommendations provided by the Society of Vascular Surgery.^11^ All aneurysm volume measurements were obtained through contrast-enhanced computed tomography (CT) imaging using semi-automatically generated center-lumen line reconstructions performed on dedicated reconstruction software (3-mensio Vascular 4.2; Pie medical, Bilthoven, The Netherlands), as previously reported and validated.^12,13^ These measurements in our cohort from previous studies exhibit an intra-observer and inter-observer variability ranging from 2% to 6%.^12,14^ Contrast-enhanced computed tomography angiography (CTA) was systematically conducted at intervals of 1, 6, and 12 months, followed by annual assessments thereafter. The aneurysm sac volume was determined from 10 mm distal to the lower-renal artery to 10 mm proximal to the aortic bifurcation.

### Definitions and Variables

Smoking status was categorized into non-smoker, current smoker, and former smoker. Patients were classified as former smoker if they had quit smoking at least 1 month prior to their surgery date. The estimated glomerular filtration rate (eGFR) was calculated using the Chronic Kidney Disease Epidemiology Collaboration (CKD-EPI 2021) equation, in accordance with the guidelines.^15^ Preoperative renal function was then further categorized into three groups based on the eGFR value: less than 30 mL/min/1.73^2^, between 30 and 60 mL/min/1.73^2^, and greater than 60 mL/min/1.73m^2^. The presence of neck seal thrombus and calcification was classified into quartiles based on the extent of circumferential involvement.

Our primary outcome included the postoperative aneurysm sac volume over time. We used the first postoperative CT scan as the baseline value for all patients, as per the guidelines.^16^ Metformin use was defined as using metformin prior to the EVAR procedure, without monitoring metformin use during follow-up. Secondary outcomes included 8-year all-cause mortality and freedom of graft-related events. A graft-related event was determined as a composite outcome and included a graft-related complication (post-implant rupture, type Ia/III/undefined endoleak, bowel/acute limb ischemia, buttocks claudication, graft infection) or procedure (proximal cuff, Palmaz stent, limb extension, iliac percutaneous transluminal angioplasty [PTA], conversion to aorto-uni-iliac configuration or open repair, coil embolization, other surgical/endovascular intervention, open/laparoscopic ligation of collaterals, fenestrated, or branched graft).^11^

### Statistical Analysis

Categorical variables were described as counts and percentages and continuous variables as means or medians, with standard deviations (SDs) or interquartile ranges (IQRs), respectively, according to their distribution. Differences in characteristics between groups were compared using Pearson’s chi-square or Fisher’s exact tests for categorical variables and Student’s *t* test or Mann-Whitney *U*-test for continuous variables, as appropriate for their distribution. Normal distribution was assessed through visual aids such as histograms and Q-Q plots or using the Shapiro-Wilk test.

Absolute changes in postoperative aneurysm sac volume and percentage changes in volume relative to the baseline measurements were studied using linear mixed-effect models. The model utilized metformin use and time as the independent variables and aneurysm sac volume as the dependent variable. To account for within-patient variability, individual patient intercepts and slopes were included. Interaction effects of metformin with time were tested and dropped when not significant. We accounted also for the potential nonlinear effect of time using quadratic terms. The time curves were fitted using the predicted values from the mixed-effects model.

In addition, we conducted a subgroup analysis focusing on patients who experienced aneurysm sac growth 1-year post-EVAR. This subgroup was analyzed using the same linear mixed-effects modeling approach previously described. The purpose of this subgroup analysis was to determine whether metformin could have a protective effect on aneurysm growth in this subgroup, which might not be apparent in the analysis of the full cohort.

Our secondary outcomes of 8-year all-cause mortality and freedom of graft-related events were assessed using Kaplan-Meier methods. If the standard error was more than 10%, the risk table or life graph was truncated. Multivariable Cox regression analysis was performed to adjust for confounding. Covariates incorporated into the linear mixed-effects models and multivariable Cox regression analyses were age, sex, smoking history, and aneurysm diameter at baseline. Confidence intervals (CIs) of 95% were used and a p value of <0.050 was considered statistically significant. Most variables had <5% missing data, unless otherwise specified. Notably, aneurysm sac volume had 23.4% and aneurysm diameter had 16.5% missing data; these cases were excluded from the linear mixed-effect model to ensure analysis accuracy. Statistical analyses were conducted using R version 4.2.2 (www.r-project.org).

## Results

### Baseline Characteristics

From 2000 to 2022, we identified 685 patients undergoing standard infrarenal EVAR for AAA of which 51 (7%) used metformin perioperatively (Figure 1). All patients on metformin were diagnosed with DM, while only a small proportion of patients without metformin had the disease (100% vs 7.3%; p<0.001; Table 1). Patients with metformin were more likely to receive concurrent treatment with beta blockers compared with those without metformin (72.5% vs 46.7%; p=0.028). The median follow-up was 4.0 years (IQR=1.5, 6.5) for metformin patients and 5.0 years (IQR=2.0, 8.0; p=0.091) for non-metformin patients.

**Table 1.** Baseline Characteristics of Patients Undergoing Endovascular Aneurysm Repair (EVAR) Stratified by Metformin Use.

	No metformin (N=634)	Metformin (N=51)	p
Age median (IQR)	73.0 (68.0, 78.0)	70.5 (67.0, 75.8)	0.260
Female sex	74 (11.7)	4 (7.8)	*0.549*
Diabetes mellitus	46 (7.3)	51 (100)	*<0.001*
Hypertension	430 (67.8)	41 (80.4)	*0.058*
Ischemic coronary disease	222 (35.0)	24 (47.1)	*0.124*
Pulmonary disease	119 (18.8)	15 (29.4)	*0.101*
Smoking status			*0.067*
*Non-smoker*	158 (24.9)	6 (11.8)	*0.059*
*Current smoker*	231 (36.4)	18 (35.3)	*1*
*Former smoker*	233 (36.8)	25 (49.0)	*0.084*
*eGFR* ^a^			*0.112*
*<30*	42 (6.6)	6 (11.8)	*0.055*
*30–60*	279 (44.0)	17 (33.3)	*0.849*
*>60*	152 (24.0)	7 (13.7)	*0.419*
Antiplatelet and/or anticoagulant^b^	470 (74.1)	43 (84.3)	*0.770*
Statin^c^	381 (60.1)	42 (82.4)	*0.185*
Beta blocker^c^	296 (46.7)	37 (72.5)	0.028

eGFR, estimated glomerular filtration rate; IQR, interquartile range.

Data were presented as counts with percentages (%) or medians with interquartile ranges (IQRs).

a Variable with <30% missing data.

b Variable with <10% missing data.

c Variable with <20% missing data.

### Anatomical/Procedural Characteristics

The proportion of aorto-iliac AAA was comparable between the groups (19.6% vs 27.4%; p=0.293; Table 2). Compared with non-metformin users, patients with metformin did not show a difference in total procedure time (105 [IQR=90, 155] vs 103 [IQR=90, 135]; p=0.823) or use of contrast (60 [IQR=53, 80] vs 70 [IQR=55, 90]; p=0.135). Prior to any exclusions, we observed no statistically significant difference between the groups in terms of urgency status (metformin vs non-metformin: elective 81.4% vs 77.0%; symptomatic 5.1% vs 9.9%; ruptured 13.6% vs 13.2%; p=0.484), however, metformin users had a smaller preoperative aneurysm sac volume (174 cc [95% CI: 123, 234] vs 184 cc [95% CI: 137, 268]; p=0.025).

**Table 2.** Anatomical and Procedural Characteristics of Patients Undergoing EVAR, Stratified by Metformin Use.

	No metformin (N=634)	Metformin (N=51)	p
Main body diameter (mm)^a^, median (IQR)	28.0 (26.0, 32.0)	28.0 (28.0, 32.0)	*0.815*
AAA volume (cc)^b^, median (IQR)	178 (133, 240)	153 (114, 195)	*0.054*
AAA diameter (mm)^c^, median (IQR)	59.0 (54.0, 67.0)	57.0 (53.0, 68.0)	*0.362*
Aneurysm type			*0.293*
Aortic AAA	460 (72.6)	41 (80.4)	*0.293*
Aorto-iliac AAA	174 (27.4)	10 (19.6)	*0.293*
Infectious AAA	10 (1.6)	1 (2.0)	*0.570*
Anastomotic/pseudoaneurysm	17 (2.7)	2 (3.9)	*0.643*
Proximal seal length (mm)^b^	21.0 (13.0, 27.0)	21.0 (12.5, 30.5)	*0.221*
Proximal neck/sealing diameter (mm)	24.0 (21.9, 26.0)	24.0 (21.3, 25.8)	*0.492*
Neck/sealing zone			
Neck length <15 mm^b^	114 (15.6)	9 (15.3)	*1.000*
Neck diameter >30 mm^c^	48 (6.6)	4 (6.8)	*0.540*
Thrombus >25%^d^	361 (49.5)	21 (35.6)	*0.375*
Calcium >25%^b^	134 (18.4)	11 (18.6)	*0.630*
Alpha angle (degrees)^b^	20.0 (11.0, 33.0)	22.0 (11.5, 32.5)	*0.879*
Beta angle (degrees)^b^	36.0 (23.0, 52.0)	34.0 (26.5, 50.5)	*0.889*
Timing of surgery			*0.331*
Elective	562 (88.6)	48 (94.1)	*0.331*
Symptomatic	72 (11.4)	3 (5.9)	*0.331*
Total procedure time (minute)^b^, median (IQR)	105 (90.0, 135)	105 (90.0, 155)	*0.823*
Contrast use (mL)^b^, median (IQR)	70.0 (55.0, 90.0)	60.0 (52.5, 80.0)	*0.135*
Fluoroscopy time (minute)^b^, median (IQR)	21.0 (14.0, 30.8)	23.0 (15.0, 37.5)	*0.333*
Blood loss (mL)^d^, median (IQR)	150 (50.0, 300)	100 (50.0, 238)	*0.301*
Intraoperative complications	78 (12.3)	5 (9.8)	*0.784*
Device modality			*0.568*
Excluder	226 (35.6)	14 (27.5)	*0.286*
Endurant	340 (53.6)	32 (62.7)	*0.245*
Talent	9 (1.4)	0 (0)	*1*
Other	55 (8.7)	5 (9.8)	*0.796*

AAA, abdominal aortic aneurysm; IQR, interquartile range.

Data were presented as counts with percentages (%) or medians with interquartile ranges (IQRs).

a Variable with <10% missing data.

b Variable with <30% missing data.

c Variable with <20% missing data.

d Variable with >30% missing data.

### Short-term Postoperative Outcomes

Metformin users were associated with similar rates of postoperative 30-day mortality compared with non-metformin users following EVAR for AAA (0.9% vs 0%; p=1.000; Table 3). Metformin users had similar rates of all types of endoleaks compared with non-metformin users. Type 2 endoleak was the most common type in both groups (15.8% vs 9.8% in non-metformin users and metformin users, respectively).

**Table 3.** Postoperative Outcomes of Patients Following EVAR, Stratified by Metformin Use.

	No metformin (N=634)	Metformin (N=51)	p
Postoperative events	190 (30.0)	9 (17.6)	*0.094*
All-cause mortality	6 (0.9)	0 (0)	*1*
Type IA endoleak	11 (1.7)	0 (0)	*1*
Type IB endoleak	2 (0.3)	0 (0)	*1*
Type II endoleak	100 (15.8)	5 (9.8)	*0.334*
Respiratory complications	11 (1.7)	0 (0)	*1*
Myocardial infarction	15 (2.4)	0 (0)	*0.619*
Renal deterioration without dialysis	12 (1.9)	0 (0)	*1*
Acute limb ischemia	12 (1.9)	1 (2.0)	*1*
Other events	30 (4.7)	3 (5.9)	*0.731*
Postoperative procedures	32 (5.0)	3 (5.9)	*0.737*
Access-related surgical procedure	13 (2.1)	0 (0)	*0.614*
Iliac PTA/stenting	5 (0.8)	0 (0)	*1*
Other surgical intervention	9 (1.4)	2 (3.9)	*0.192*
Other endovascular interventions	3 (0.5)	2 (3.9)	*0.046*

PTA, percutaneous transluminal angioplasty.

Data were presented as counts with percentages (%).

### Postoperative Aneurysm Sac Volume Over Time

**Figure 2.**
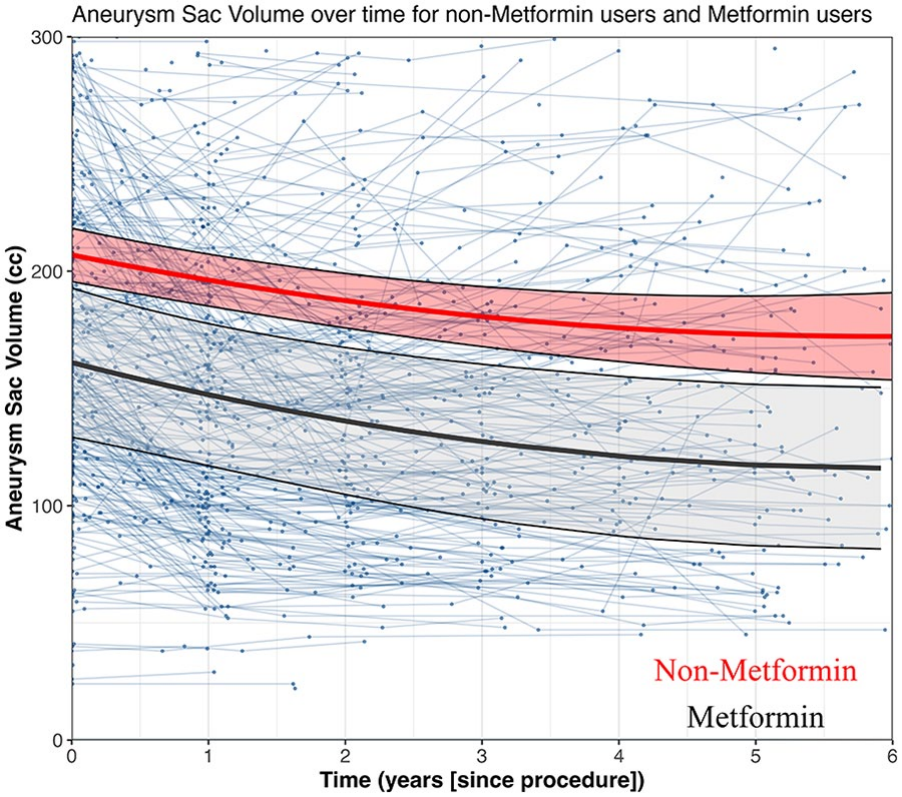
Linear mixed-effects model displaying absolute aneurysm sac volume (cc) over time for no metformin and metformin use.

Figure 2 illustrates the unadjusted longitudinal mixed-effects model-derived time curves for absolute aneurysm sac volume as a function of time. The adjusted model indicated that at baseline, 30 days after EVAR, the mean aneurysm sac volume was comparable between the groups (metformin vs non-metformin: –19.4 cc; [95% CI: –47.4, 8.5]; p=0.173; Supplementary Table 1). The results revealed a significant nonlinear shrinkage of the aneurysm sac volume over time (time: –17.4 cc/year; [95% CI: –21.4, –13.4] and time^2: 2.0 cc/year^2; [95% CI: 1.6, 2.5]; p<0.001). While metformin use was associated with a trend toward reduced aneurysm volume, the interaction terms between metformin and time did not reach statistical significance (metformin×time: –3.9 cc/year; [95% CI: –22.7, 14.9]; p=0.685). Relative to the baseline measurements, aneurysm volume decreased over time as well (time: –8.6%/year; [95% CI: –10.4, –6.7]; p<0.001), but the interaction terms between metformin and time were not significant (metformin×time: –3.8%/year; [95% CI: –9.5, 1.9]; p=0.192).

In our subgroup analysis, which focused on patients demonstrating aneurysm sac growth 1 year post-EVAR, we observed a significant linear expansion of the aneurysm sac volume over time (time: 19.4 cc/year; [95% CI: 3.98, 34.8]; p<0.001). However, this expansion appeared to be unrelated to metformin use, as indicated by the non-significant interaction terms between metformin and time (metformin×time: –12.6 cc/year; [95% CI: –75.7, 50.5]; p=0.699).

Relative to the baseline measurements, aneurysm volume decreased over time as well (time: 10.0%/year; [95% CI: 3.7, 16.3]; p=0.003), but the interaction terms between metformin and time were not significant (metformin×time: –4.0%/year; [95% CI: –29.6, 21.5]; p=0.756). These results indicate that metformin does not appear to have a substantial effect on the postoperative aneurysm sac volume over time.

### Long-term events

**Figure 3.**
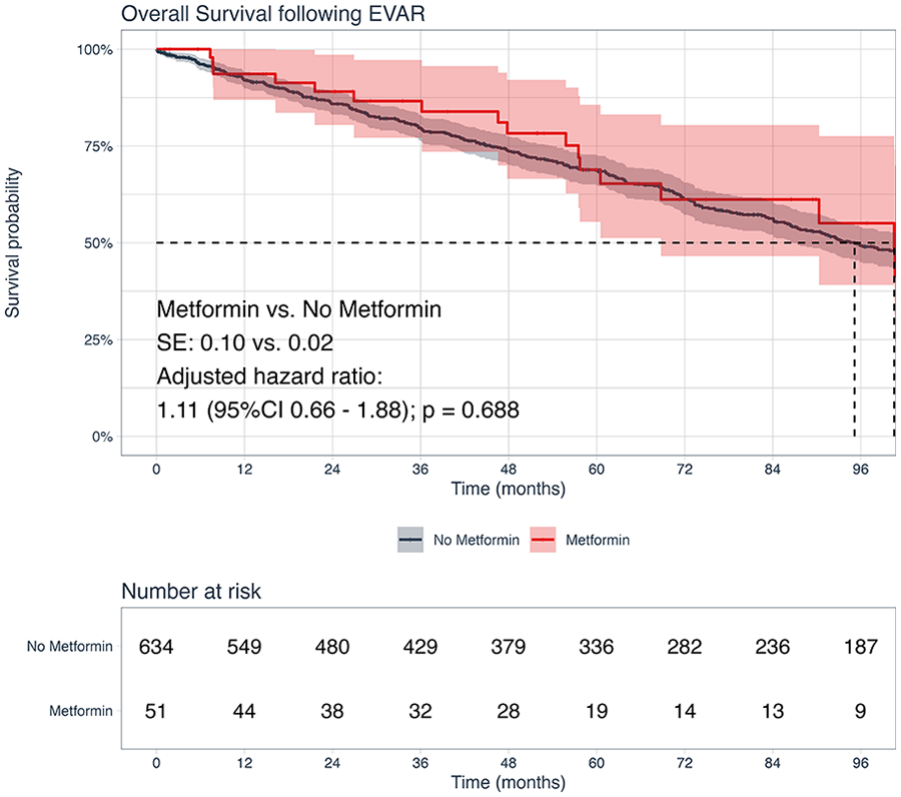
Kaplan-Meier analysis for freedom from all-cause mortality stratified by perioperative metformin use. Caption: standard errors were below 10% at all time-points; highlighted area marks 95% confidence intervals.

After risk adjustment, metformin was associated with similar 8-year all-cause mortality risk (metformin vs no metformin: 50% vs 44%; hazard ratio [HR]=1.11, 95% CI: 0.66, 1.88; p=0.688; Figure 3). Other factors, including age (HR=1.07, 95% CI: 1.05, 1.08; p<0.001) and smoking (HR=1.30, 95% CI: 1.01, 1.67; p=0.044) were associated with a higher risk of all-cause mortality.

**Figure 4.**
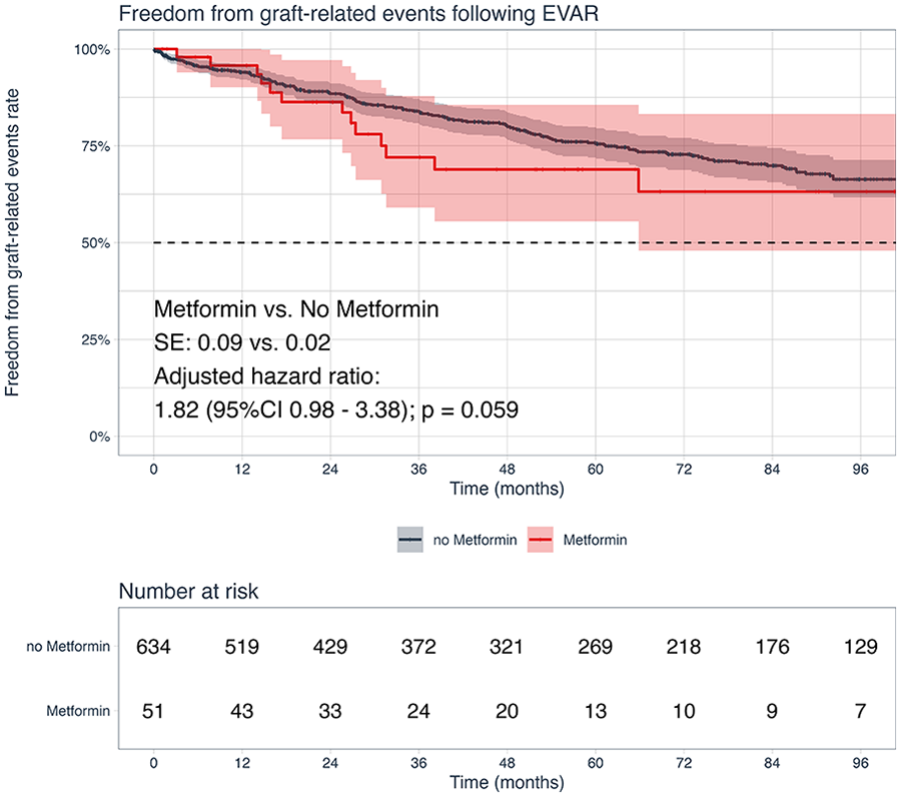
Kaplan-Meier analysis for freedom from graft-related events stratified by perioperative metformin use. Caption: standard errors were below 10% at all time-points; highlighted area marks 95% confidence intervals.

Following risk adjustment, metformin use was also associated with a comparable risk of 8-year freedom from graft-related events risk (metformin vs no metformin: 63% vs 66%; HR=1.82, 95% CI: 0.98, 3.38; p=0.059; Figure 4). The details of the graft-related events are shown in Table 4. In addition, age (HR=0.98, 95% CI: 0.95, 1.00; p=0.036) was found to be associated with a decreased risk of graft-related events. We found no significant differences in the estimates of freedom from endoleak type I (p=0.500) and type II (p=0.093; Supplementary Figures 1 and 2).

**Table 4.** Details of Graft-Related Events Following EVAR, Stratified by Metformin Use.

	No metformin (N=634)	Metformin (N=51)	p
Follow-up (months)	63.0 (26.0, 105)	52.0 (23.0, 80.0)	*0.074*
Graft-related event(no. of patients)	162 (25.6)	15 (29.4)	0.180*
Graft-related complication, patients (n)	90 (14.2)	9 (17.6)	0.001*
Post-implant rupture	6 (0.9)	1 (2.0)	
Secondary endoleak			*0.584*
Type Ia	41 (6.5)	4 (7.8)	*0.799*
Type III	6 (0.9)	1 (2.0)	*0.586*
Undefined endoleak	9 (1.4)	1 (2.0)	*1*
Bowel/acute limb ischemia	20 (3.2)	4 (7.8)	*0.502*
Buttocks claudication	11 (1.7)	0 (0)	*0.373*
Graft infection	6 (0.9)	0 (0)	*1*
Secondary procedure, patients (n)	139 (21.9)	12 (23.5)	0.023*
Proximal cuff	17 (2.7)	1 (2.0)	*0.692*
Palmaz stent	5 (0.8)	1 (2.0)	*0.520*
Limb extension	46 (7.3)	4 (7.8)	*0.589*
Iliac PTA	11 (1.7)	1 (2.0)	*1*
Conversion to AUI or open repair	24 (3.8)	3 (5.9)	*1*
Coil embolization	10 (1.6)	0 (0)	*0.600*
Fenestrated or branched graft	18 (2.8)	1 (2.0)	*0.694*
Open or laparoscopic ligation of collaterals	10 (1.6)	0 (0)	*0.600*
Surgical/ endovascular intervention^†^	39 (6.2)	5 (9.8)	*1*

PTA, percutaneous transluminal angioplasty; AUI, aorto-uni-iliac configuration.

* Calculated using log-rank analysis.

† Surgical/endovascular interventions included thrombectomy, embolization, bypass surgeries, and specific graft modifications.

Data were presented as medians with interquartile ranges (IQRs) or as counts with percentages (%).

## Discussion

In this retrospective cohort study including 685 patients, we aimed to investigate the impact of metformin use on treatment and long-term outcomes following EVAR for infrarenal AAA. Initially, our observations indicated that metformin and non-metformin patients presented with comparable proportions of ruptured aneurysms, however, those on metformin were at smaller aneurysm volumes. Analysis of postoperative aneurysm sac volume over time showed no significant effect of metformin on aneurysm growth over time following EVAR. In addition, metformin users had similar rates of 8-year all-cause mortality compared with non-metformin users. Finally, metformin use was associated with a similar risk of graft-related events.

Our analysis did not demonstrate a significant impact of metformin on both absolute and relative aneurysm sac volume over time following EVAR. We found no significant difference in baseline aneurysm volume and the course of the aneurysm sac volume over time was parallel between the groups. This finding contrasts with previous research suggesting a suppressive effect of metformin on AAA growth. For instance, Fujimura et al^1^ found a negative association between metformin use and AAA enlargement, while Unosson and colleagues reported significantly lower AAA growth in diabetic patients taking metformin compared with non-diabetic patients.^17^ Moreover, Itoga et al^18^ demonstrated that metformin use was associated with an additional reduction in AAA enlargement, beyond that which could be attributed to diabetes alone. We did not observe such associations in our post-EVAR analyses. The surgical intervention might have altered the natural course of AAA growth, thus limiting the potential for metformin to affect the aneurysm sac volume. Furthermore, larger AAAs have been associated with increased rates of mortality, reinterventions and ruptures following EVAR.^19^ The potential of metformin to limit aneurysm growth could therefore offer significant benefits for patient outcomes. Nevertheless, in our subgroup analysis focused on patients who experienced aneurysm growth post-EVAR, we did not observe a significant association between metformin use and aneurysm growth.

Our study did not find a significant effect of metformin use on 8-year all-cause mortality. This contrasts with previous research, wherein metformin use, along with EVAR, was associated with a reduced risk of AAA-repair-related mortality after adjusting for various confounders. This beneficial effect of metformin was attributed to its anti-inflammatory properties.^20^ Moreover, Yuan et al^21^ reported that metformin could alleviate the incidence of events including death. Han et al indicated a potential reduction in cardiovascular and all-cause mortality among coronary artery disease patients due to metformin.^22^

It is important to note that the majority of studies have found an association between DM and increased mortality rates following endovascular AAA repair.^3,23–25^ In our study, the observed association of comparable mortality rates with metformin use could subtly suggest that metformin might have a protective quality. This potential benefit of metformin may counteract the inherent increased cardiovascular mortality risk associated with DM. Nevertheless, the specific mechanisms linking metformin with AAA remain under-explored. Future analyses with larger prospective samples should determine whether metformin use has an effect on long-term mortality following EVAR.

There are conflicting findings regarding the impact of metformin on graft-related events in AAA patients. Turowicz et al discovered that metformin treatment was not a significant factor affecting AAA-repair-related complications after adjusting for various risk factors among all patients.^20^ In contrast, Golledge et al reported that diabetic patients on metformin had a lower incidence of AAA events compared with those without diabetes (HR=0.63; 95% CI: 0.44, 0.93). However, diabetic patients not using metformin did not exhibit a reduced incidence of AAA events compared with non-diabetic patients.^26^ In their study, AAA events were defined as the combined incidence of AAA repair or mortality due to AAA rupture. The discrepancy in findings between the studies may be attributed to the inclusion of mortality from AAA rupture in the outcome measure of the latter study. We hypothesize that factors other than metformin use may have a more substantial effect on graft-related events. As Turowicz et al confirmed, the number of complications was more dependent on factors, such as aneurysm rupture than metformin usage.^20^ Our similar findings may be attributed to the exclusion of patients with ruptured aneurysms from our study.

The question of whether metformin should be adopted as a standard treatment option for patients with infrarenal AAA undergoing EVAR remains open for further investigation and will need a prospective randomized study. While our study has provided valuable insights, more comprehensive research is essential for drawing definitive conclusions. The ongoing MAAAGI (Metformin for Abdominal Aortic Aneurysm Growth Inhibition) trial in Sweden, which evaluates the effect of metformin on AAA growth in patients with small aneurysms, may shed light on the potential benefits of this drug in preventing AAA progression.^8^ Nevertheless, there remains a need for a well-designed randomized-controlled trial (RCT) that specifically examines post-EVAR outcomes in patients treated with metformin. Such a trial would help determine the true clinical impact of metformin use in the context of EVAR and clarify its role as a standard treatment option for patients with infrarenal AAA.

When interpreting this study, several key limitations should be acknowledged. First, the retrospective nature of the study design may introduce potential selection bias and limit our ability to establish causality between metformin use and post-EVAR outcomes. Numerous anatomical and demographic characteristics could influence aneurysm sac dynamics following EVAR. All patients on metformin in this study had DM. However, potential confounders, such as the presence of DM and concurrent treatment with statins, were not controlled for in the analysis. Given the low incidence of events, the statistical power to perform multivariate analyses was limited. Consequently, we may have been unable to differentiate between the effects of metformin and the presence of DM. While metformin was prescribed to control DM, its effect on aneurysm sac dynamics might be linked with, or possibly enhanced by, the underlying DM. Second, these findings must be interpreted with caution, due to potential limitations in statistical power. The relatively small sample size of metformin users (n=51) compared with non-metformin users (n=634) may reduce the ability to detect significant differences between these groups, potentially contributing to type II errors. A retrospective study without a prior power analysis is always at risk for type II errors. Nevertheless, our study represents the largest sample size to date investigating the association between metformin use and long-term outcomes after EVAR. Furthermore, the single-center setting may limit the generalizability of the results to other institutions or populations. Moreover, the registration of graft-related events primarily depended on the number and timing of postoperative CTAs. Although we attempted to correct for this using a composite outcome, this limitation remains a source of information bias. Finally, data on long-term metformin use and adherence were not available, due to the retrospective nature of the study. Patients might have changed their use of metformin postoperatively, either discontinuing or initiating metformin use. This could have introduced non-differential misclassification bias into our results, potentially limiting the effect of metformin on aneurysm growth over time. If the MAAAGI trial shows any positive effects on limiting growth rate before treatment, it would be sensible to analyze this in a prospective manner after AAA treatment.^8^ Despite these limitations, this study offers valuable insights into the association of metformin with long-term postoperative outcomes following EVAR for infrarenal AAA and contributes to the ongoing debate on the role of metformin in patients with AAA.

## Conclusion

Among patients undergoing EVAR for AAA, metformin use was associated with similar aneurysm sac dynamics/volume over time compared with non-metformin use. Furthermore, metformin use was associated with similar perioperative and 8-year mortality as well as freedom from graft-related events. This suggests that the potential protective effect of metformin on AAA may not be sustained after EVAR. These findings highlight the need for further studies to examine the underlying mechanisms of the potential role of metformin in AAA management following EVAR.

## Supplemental Material

sj-docx-1-jet-10.1177_15266028241268500 – Supplemental material for Metformin Use and Long-term Outcomes Including Aneurysm Sac Dynamics Following EVAR for Infrarenal Abdominal Aortic Aneurysm: “A Retrospective Study” Supplemental material, sj-docx-1-jet-10.1177_15266028241268500 for Metformin Use and Long-term Outcomes Including Aneurysm Sac Dynamics Following EVAR for Infrarenal Abdominal Aortic Aneurysm: “A Retrospective Study” by Olivier L. R. M. van Tongeren, Vinamr Rastogi, David E. Vecht, Klaas H. J. Ultee, Sanne E. Hoeks, Hence J. M. Verhagen and Jorg L. de Bruin in Journal of Endovascular Therapy

sj-tiff-2-jet-10.1177_15266028241268500 – Supplemental material for Metformin Use and Long-term Outcomes Including Aneurysm Sac Dynamics Following EVAR for Infrarenal Abdominal Aortic Aneurysm: “A Retrospective Study” Supplemental material, sj-tiff-2-jet-10.1177_15266028241268500 for Metformin Use and Long-term Outcomes Including Aneurysm Sac Dynamics Following EVAR for Infrarenal Abdominal Aortic Aneurysm: “A Retrospective Study” by Olivier L. R. M. van Tongeren, Vinamr Rastogi, David E. Vecht, Klaas H. J. Ultee, Sanne E. Hoeks, Hence J. M. Verhagen and Jorg L. de Bruin in Journal of Endovascular Therapy

sj-tiff-3-jet-10.1177_15266028241268500 – Supplemental material for Metformin Use and Long-term Outcomes Including Aneurysm Sac Dynamics Following EVAR for Infrarenal Abdominal Aortic Aneurysm: “A Retrospective Study” Supplemental material, sj-tiff-3-jet-10.1177_15266028241268500 for Metformin Use and Long-term Outcomes Including Aneurysm Sac Dynamics Following EVAR for Infrarenal Abdominal Aortic Aneurysm: “A Retrospective Study” by Olivier L. R. M. van Tongeren, Vinamr Rastogi, David E. Vecht, Klaas H. J. Ultee, Sanne E. Hoeks, Hence J. M. Verhagen and Jorg L. de Bruin in Journal of Endovascular Therapy
