# Supplementary material for: Metformin Use and Long-term Outcomes Including Aneurysm Sac Dynamics Following EVAR for Infrarenal Abdominal Aortic Aneurysm: “A Retrospective Study”
Source: J Endovasc Ther. 2024 Aug 14;33(2):749–58. doi: 10.1177/15266028241268500 (PMC12972099; doi:10.1177/15266028241268500)
Supplement: sj-docx-1-jet-10.1177_15266028241268500 – Supplemental material for Metformin Use and Long-term Outcomes Including Aneurysm Sac Dynamics Following EVAR for Infrarenal Abdominal Aortic Aneurysm: “A Retrospective Study” [file sj-docx-1-jet-10.1177_15266028241268500.docx]

| Supplementary Table 1. Linear Mixed-effects model results for absolute aneurysm sac volume (cc) over time for all patients. | | | | |
| --- | --- | --- | --- | --- |
| Predictor Variable | Coefficient | SE | t-value | *P-value* |
| Metformin (vs. No Metformin) | - 19.4 | 14.2 | -1.4 | *0.173* |
| [Time] (years) | - 17.4 | 2.0 | -8.5 | *<0.001* |
| [Time]^2 (years) | 2.0 | 0.2 | 8.8 | *<0.001* |
| Constant | -229.3 | 35.0 | -6.5 | *<0.001* |
| *SE,* standard error. | | | | |
