# Supplementary figures and images for: Metformin Use and Long-term Outcomes Including Aneurysm Sac Dynamics Following EVAR for Infrarenal Abdominal Aortic Aneurysm: “A Retrospective Study”
Source: J Endovasc Ther. 2024 Aug 14;33(2):749–58. doi: 10.1177/15266028241268500 (PMC12972099; doi:10.1177/15266028241268500)

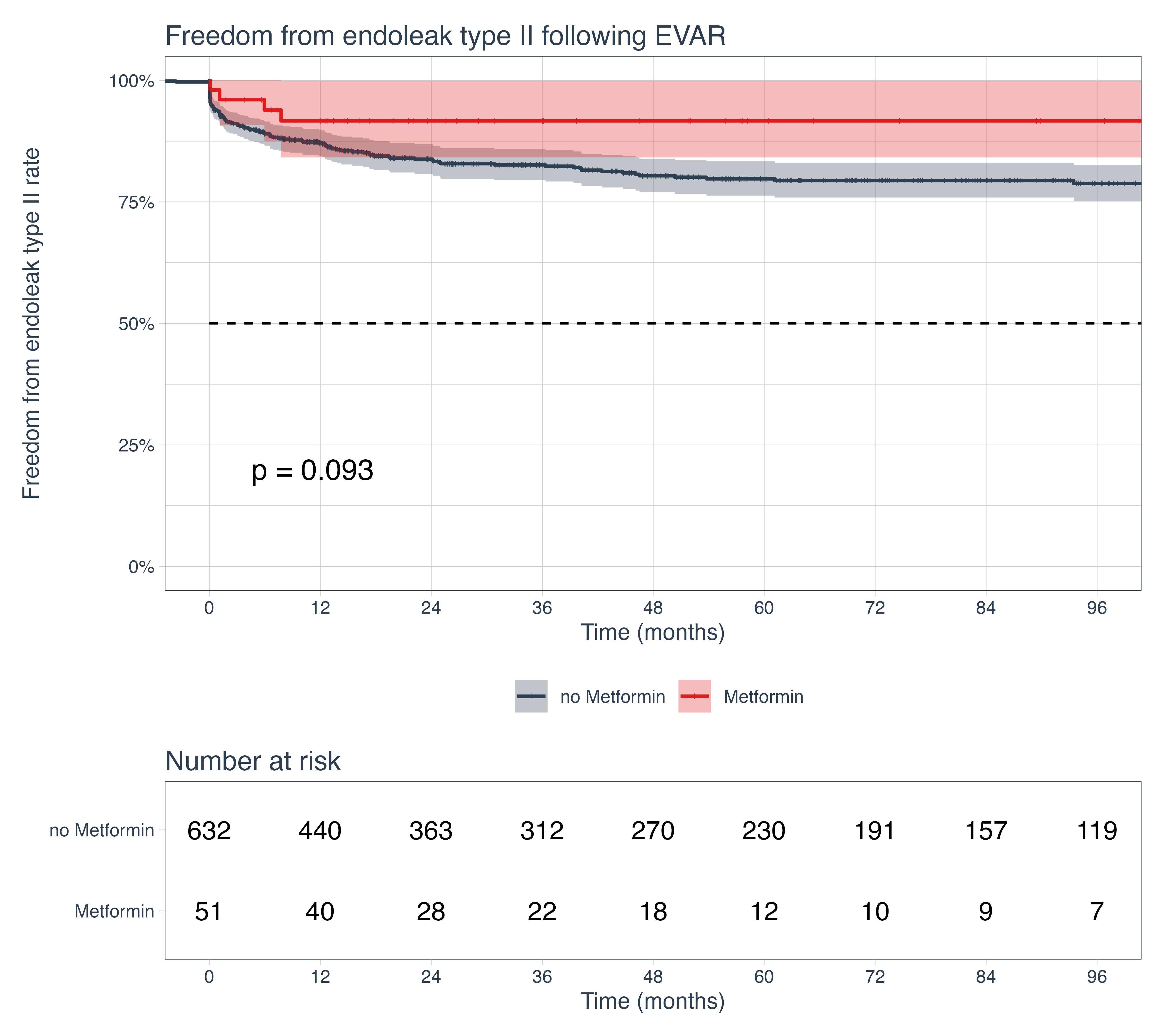

Supplement: sj-tiff-2-jet-10.1177_15266028241268500 – Supplemental material for Metformin Use and Long-term Outcomes Including Aneurysm Sac Dynamics Following EVAR for Infrarenal Abdominal Aortic Aneurysm: “A Retrospective Study” [file sj-tiff-2-jet-10.1177_15266028241268500.tiff]

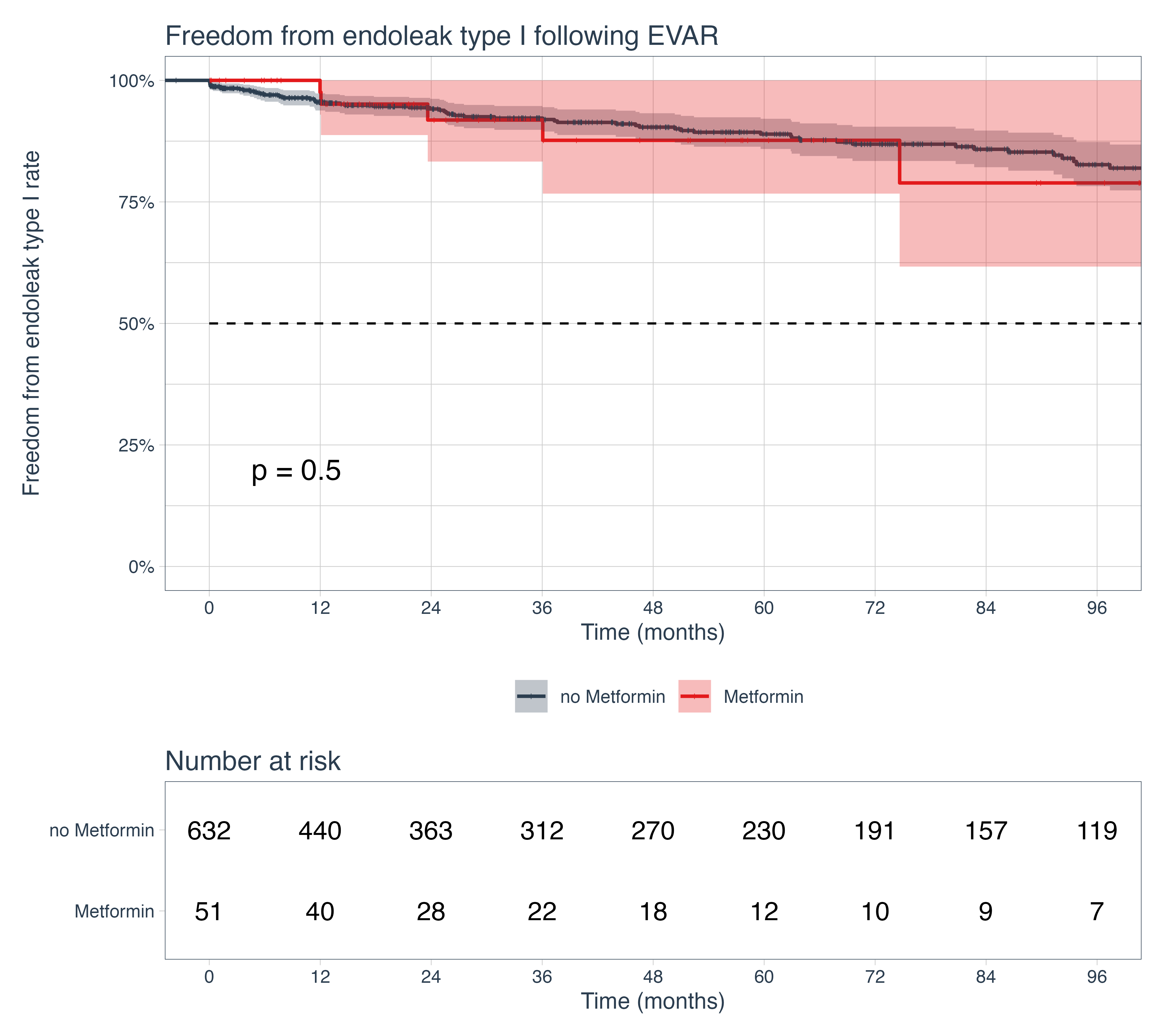

Supplement: sj-tiff-3-jet-10.1177_15266028241268500 – Supplemental material for Metformin Use and Long-term Outcomes Including Aneurysm Sac Dynamics Following EVAR for Infrarenal Abdominal Aortic Aneurysm: “A Retrospective Study” [file sj-tiff-3-jet-10.1177_15266028241268500.tiff]
